# Rationally Engineered Synthetic Coculture for Improved Biomass and Product Formation

**DOI:** 10.1371/journal.pone.0113786

**Published:** 2014-12-03

**Authors:** Suvi Santala, Matti Karp, Ville Santala

**Affiliations:** Department of Chemistry and Bioengineering, Tampere University of Technology, Tampere, Finland; Tsinghua University, China

## Abstract

In microbial ecosystems, bacteria are dependent on dynamic interspecific interactions related to carbon and energy flow. Substrates and end-metabolites are rapidly converted to other compounds, which protects the community from high concentrations of inhibitory molecules. In biotechnological applications, pure cultures are preferred because of the more straight-forward metabolic engineering and bioprocess control. However, the accumulation of unwanted side products can limit the cell growth and process efficiency. In this study, a rationally engineered coculture with a carbon channeling system was constructed using two well-characterized model strains *Escherichia coli* K12 and *Acinetobacter baylyi* ADP1. The directed carbon flow resulted in efficient acetate removal, and the coculture showed symbiotic nature in terms of substrate utilization and growth. Recombinant protein production was used as a proof-of-principle example to demonstrate the coculture utility and the effects on product formation. As a result, the biomass and recombinant protein titers of *E. coli* were enhanced in both minimal and rich medium simple batch cocultures. Finally, harnessing both the strains to the production resulted in enhanced recombinant protein titers. The study demonstrates the potential of rationally engineered cocultures for synthetic biology applications.

## Introduction

In a microbial ecosystem, bacterial species cooperate by producing metabolites that serve as substrates for other species of a community. Dynamic and symbiotic interactions between different species enable an efficient carbon and energy flow. Furthermore, the interactions balance rapid environmental changes and high concentrations of substrates, intermediates, or other potentially harmful compounds. Common end-metabolites, such as organic acids, are rapidly converted to other compounds. Thus, tolerance to high concentrations of inhibitory compounds is not essential for individual members of a community. In biotechnological applications, however, engineered pure cultures are typically utilized as production platforms because of the easier bioprocess control and more straight-forward genetic engineering. Nevertheless, establishing a sustainable and economical bioprocess is a challenge, for which metabolic engineering and synthetic biology are seeking solutions [1]–[4]. For example, accumulation of unwanted side products and inefficient carbon and energy fluxes are common issues. Since *Escherichia coli* is a widely used host in bioprocesses, considerable effort is dedicated to controlling the overflow metabolism. In this phenomenon, carbon is excessively transported into the cells, and the capacity of tricarboxylic acid (TCA) cycle is exceeded. As a result, the metabolism is shifted to produce acetate from acetyl coenzyme A (CoA) [5]. The accumulation of acetate results in the induction of the stress response, growth inhibition, significant carbon loss, and eventually reduced productivity [6], [7].

Previous strategies to neglect the negative effects of acetate include both genetic engineering of *E. coli* to circumvent the acetate production and comprehensive optimization of fed-batch bioprocesses [8]–[10]. These approaches, however, potentially decrease the growth rate or require a substantial amount of optimization and engineering work. “Pure cocultures” typically consisting of two known strains are a more recent approach to improve the production platforms [11], [12], and a trend towards more controllable and tunable cocultures involving systems biology and synthetic biology solutions is growing stronger [13], [14]. For example, Eiteman et al. [15] have described the co-fermentation of hexoses and pentoses by a community of engineered *E. coli.* Tsai et al. [16] established a system for ethanol production from cellulose by a synthetic yeast community. Although the obtained ethanol yields are low, the study is an elegant example of the utilization of an engineered community for bioenergy production.

The culture systems described above are based on exploiting strains of single species, but they represent the first steps towards consolidated processing of sustainable substrate to products by an engineered consortium. Employing different strains of a single species broadens the engineering possibilities; however, the strains often possess very similar carbon utilization patterns, thus leaving unresolved the issues related to by-product accumulation and the inefficient carbon flow. On the other hand, genetic engineering is very challenging for mixed populations which efficiently degrade complex substrates and mixed sugars [17]. In the present study, the advantages of both mixed populations and readily engineered model strains are exploited; the well-characterized model organisms *E. coli* K12 and *Acinetobacter baylyi* ADP1 were employed for the construction of a synthetic and symbiotic microecosystem with directed carbon flow for improved culture performance. In the first stage of the study, the strains were cultured in a minimal medium batch cultivation to monitor the coculture compatibility, performance of individual strains, and carbon flow. Secondly, *E. coli* strain was engineered to produce recombinant protein, and batch cocultures with different substrate concentrations were conducted to demonstrate the effects of accelerated growth to recombinant protein production. Finally, both strains were engineered to produce the same recombinant protein, and cocultivations in a bioreactor were carried out.

## Materials and Methods

### Strains

In the initial coculture, *E. coli* K12 BW25113 (from Yale *E. coli* Genetic Stock Center CGSC, Connecticut, USA) was used. The single gene knock-out strain of *A. baylyi* ADP1 (DSM 24193) (gene deletion high-affinity gluconate permease, *gntT*, ACIAD0544) was employed in the study (kindly provided by Dr. Veronique de Berardinis, Genoscope, France). In the single gene knock-out mutant, the target gene is replaced with a gene cassette containing a kanamycin resistance gene (*Kan^r^*) [18]. The strain ADP1Δ*gnt::Kan^r^/tdk* was transformed with a construct pVKK81-T-*lux*, and designated as ABlux. In the first stage of GFP production, *E. coli* K12 BW25113 was transformed with sfGFP/pAK400c and designated as ECsf. In the second stage, both *E. coli* K12 BW25113 and *A. baylyi* ADP1Δ*gnt::Kan^r^/tdk* were transformed with a plasmid pBAV1C-T5-GFP, resulting in strains designated as ECg and ABg, respectively. For monitoring the individual GFP production of *E. coli*, a control strain ADP1Δ*gnt::Kan^r^/tdk* transformed with a plasmid pBAV1C-*ara* without the insert was constructed, and the strain was designated as ABc. Genetic constructs are described in the section “plasmid construction and transformations”. The same strains without the plasmids were used as background control strains for fluorescence measurement.

### Plasmid construction and transformations

For digestions and ligations, the enzymes and buffers were provided by Fermentas (Lithuania) and used according to the manufacturer's instructions. The PCR reagents were provided by Finnzymes (Finland) (DNA polymerase PhusionTM and buffer) and Fermentas (nucleotides). The primers were obtained from ThermoFisher Scientific (Germany) with appropriate restriction sites.

The superfolder GFP variant (from BBa_I746909) was amplified with primers 5′-GAGTTCTAGAGAAGGAGATATACATATGCGTAAAGGCGAAGAGCTGTTC-3′ and 5′-CTAGCAAGCTTAGTGGTGATGGTGATGATGTTTGTATAG-3′ with restriction sites *Xba*I and *Hind*III, respectively, and cloned to pAK400c vector [19], [20] under the IPTG-inducible lactose promoter, resulting in construct sfGFP/pAK400c. To construct the plasmid pBAV1C-T5-GFP with chloramphenicol resistance gene *cat*, the original plasmid pBAV1K-T5-GFP [21] was partially amplified with PCR using primers 5′-TAATAGCTAGCTATTTAAAGATACCCCAAGAAGCTAATTATAAC-3′ and 5′-TAATAGTGCACTCGCTTGGACTCCTGTTGATAG-3′. The product was ligated with *cat* gene amplified from pAK400c [19] using primers 5′-AATAGCTAGCCTGTAGAAAAGAGGAAGGAAATAATAAATGGAGAAAAAAATCACTGGATATAC-3′ and 5′-TAATAGTGCACTTACGCCCCGCCCTGCCAC-3′ with restriction sites *Nhe*I and *Apa*LI. For expression of bacterial luciferase operon, a plasmid pVKK81-T-*lux* (V. Santala and M. Karp, unpublished data) containing the bacterial luciferase genes *luxCDABE* from *Photorhabdus luminescens* and tetracycline resistance gene (T) was used. For ADP1, the plasmids pVKK81-T-*lux*, pBAV1C-T5-GFP, and pBAV1C-*ara* without an insert [22] were naturally transformed by the method described previously [23], and the colonies were selected from Luria-Agar (LA) plates (1 g/l NaCl, 5 g/l yeast extract, 10 g/l tryptone, 15 g/l agar) containing tetracycline (10 µg/ml) and chloramphenicol (25 µg/ml), respectively. For *E. coli*, the plasmids sfGFP/pAK400c and pBAV1C-T5-GFP were transformed by electroporation and the colonies were selected from LA plates containing chloramphenicol (25 µg/ml). The constructs were verified by restriction analysis, fluorescence, or luminescence determination.

### Medium composition and cultivations

For the initial coculture, minimal salts medium MA/9 described previously (9) was used. Glucose (∼50 mM) was used as the initial carbon and energy source. The strains were cultivated in batch bottles (50 ml medium/250 ml Erlenmeyer flasks) at 37°C and 300 rpm both individually and in cocultures for 12 h. Two parallel bottles were used for each culture.

For study of the GFP production of *E. coli* both in monoculture and in coculture at variable substrate concentrations, the strains ECg and ABc were cultivated in 5 ml of a MA/9 medium supplemented with 50 mM, 100 mM, or 250 mM glucose and 100 µM IPTG at 30°C and 300 rpm. The cultivations were performed both individually and in cocultures in two parallel tubes for 24 h.

For 0.5-litre batch cultivations in a bioreactor, the monoculture (ECg) and the cocultures (ECg and ABg or ECg and ABc) were cultivated in a modified Luria-Bertani medium (1 g/l NaCl, 5 g/l yeast extract, 10 g/l tryptone) supplemented with 2 mM MgSO_4_, 0.5 mM CaCl_2_, 3 µM FeCl_3_ and 100 mM glucose, and 25 µg/ml chloramphenicol. The cultivation was carried out in a 1-litre vessel (Sartorius Biostat B plus Twin System, Germany) with an online pH monitoring system at 30°C for 10 h. The cultures were aerated by stirring (350 rpm) and supply of oxygen (O_2_ partial pressure set to 20%). Equal inoculum sizes for each strain were used. Control cultivations without glucose supplementation were conducted in 250 ml batch bottles in otherwise similar conditions.

### Luminescence, fluorescence, and biomass determination

For luminescence measurements, samples were taken from the batch cultivations every hour and 200 µl of a sample (diluted when necessary) was applied on white microtiter well plates in three parallel samples. The luminescent signal was measured immediately with a Victor 2 plate reader (Perkin Elmer Life Sciences, Finland).

The total biomass in the bottles and in the bioreactor was determined by optical density measurement at the wavelength 600 nm every hour (OD_600_). Also, in the initial coculture and in the bioreactor experiments, the cells were plated every two hours (starting from 2 or 5 h) on LA plates with and without kanamycin (30 µg/ml) in order to determine the individual cell numbers of *E. coli* and *A. baylyi* according to CFU (colony forming units). For bioreactor batch cultures, 40 ml biomass samples were taken at the end of the cultivation, centrifuged at 30,000 g for 30 min and freeze-dried for gravimetric dry cell weight (CDW) determination.

For fluorescence determination, 1 ml samples were taken from all cultivations, centrifuged for 1 min at 10,000 g, and suspended in 1 ml 10 mM Tris-HCl buffer (pH 8.2) containing 150 mM NaCl. The samples for fluorescence measurements were diluted to linear range and measured as four 10–50 µl parallel samples with Fluoroskan Ascent FL (Thermo Labsystems, Finland) using the wavelengths 485 nm (excitation) and 538 nm (emission).

### Analysis for glucose and end-metabolite concentrations

Glucose consumption and acetate accumulation/consumption in the cultivations were determined with high performance liquid chromatography (HPLC) as described previously [24]. Identification and quantification of glucose and end-metabolites were based on co-chromatography using external standards. The mediums were used as controls.

## Results and Discussion

### Establishing a synthetic coculture

In natural ecosystems, different species interact through diverse forms of cooperation, such as mutualism, parasitism, or symbiosis, creating a complex network of integrated metabolic pathways. From a synthetic biology point of view, cocultures can provide several advantages over monocultures by performing multi-step tasks and being more catabolically versatile [25]–[27].

In this study, a synthetic coculture was constructed using two strains of different species, *E. coli* K12 and *A. baylyi* ADP1, and the effects of connecting the carbon metabolism of these strains were investigated. For the construction of a functional, synthetic coculture, the bacterial species for the community were chosen based on characteristics supporting one another. To promote straight-forward system design and engineering, the availability of readily applicable engineering tools and well-characterized metabolic networks was considered essential here [28], [29]. In microbiological terms, the strains also share similar preferences for growth conditions.

*A. baylyi* ADP1 has been recently introduced as a competitive model organism for genetic studies [18] and metabolic engineering purposes [22], [23], and genetic tools are widely available for the strain [21], [30], [31]. *A. baylyi* ADP1 exhibits a relatively wide substrate range and is known to efficiently utilize organic acids such as acetate as a sole carbon and energy source [32], although the detailed mechanism for the uptake and utilization of acetate has not yet been characterized. Moreover, our recent studies suggest that ADP1 neither produces harmful overflow metabolites nor exhibits substrate inhibition (S. Santala and V. Santala, unpublished data).

**Figure 1:**
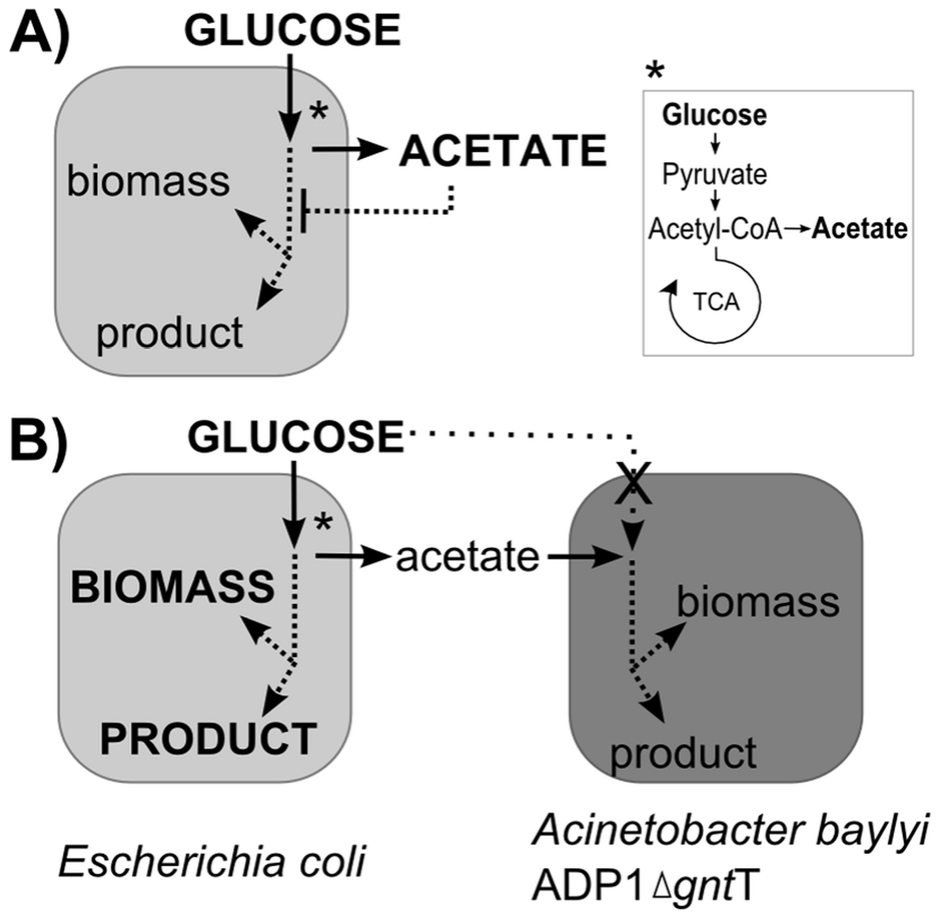
The proposed carbon flow in the wild type *Escherichia coli* culture and in the coculture of engineered *Acinetobacter baylyi* ADP1 and *E. coli*. A) *E. coli* culture supplied with excess glucose readily shifts to an overflow metabolism (*), producing large amounts of acetate into the culture medium. Acetate inhibits growth and reduces product formation, and carbon flow is directed off the product route. B) In a coculture involving directed carbon flow, the strain *A. baylyi* ADP1 is made deficient in glucose utilization by a knock-out of gluconate permease *gntT* and is solely dependent on the end-metabolites (acetate) of *E. coli*. Carbon is further metabolized and can be directed to biomass and the product of interest. Metabolic pathways in the figure are simplified and only the main products are shown.

In order to establish a functional symbiotic coculture system with directed carbon flow, and to produce unambiguous data for analyzing the coculture performance, a mutant strain of *A. baylyi* ADP1 made deficient of utilizing glucose was employed; according to the metabolic model of ADP1, the disruption of a high-affinity gluconate permease (*gntT*, ACIAD0544) blocks the glucose pathway. Due to the exceptional glucose utilization pathway of ADP1, a glucose molecule is oxidized to gluconate on the outer surface of the inner membrane by an electron carrier associated to glucose dehydrogenase, pyrroloquinoline quinine (PQQ). Subsequently, the high-affinity gluconate permease GntT transports gluconate into cells [32]. Here, a knock-out mutant strain *A. baylyi* ADP1Δ*gntT*::*Kan^r^/tdk* [18] was employed. When glucose is sufficiently available in the coculture, *E. coli* cells uptake glucose into the cells in excess and return a significant amount of the carbon into the medium in the form of acetate. Thus, in a coculture, the glucose negative mutant strain of ADP1 is dependent on the end-metabolites of *E. coli,* enabling an experimental validation of the carbon flow and fate. A schematic illustration of the proposed carbon flow is presented in Figure 1.

**Figure 2:**
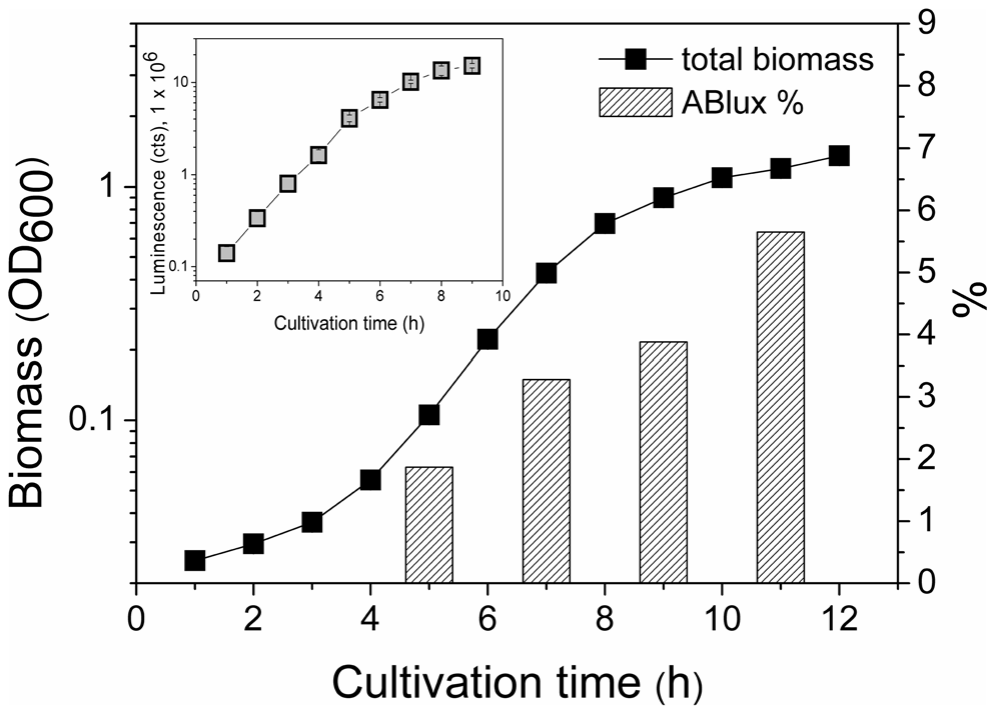
The total biomass of the coculture of *E. coli* and ADP1Δ*gntT*::*Kan^r^/tdk*[*lux_tet^r^*] (ABlux), and the proportion of ABlux in the total biomass. The strain ABlux is deficient in utilizing glucose. During the cultivation, the growth of ABlux was monitored in real-time via a luminescence reporter *luxCDABE* (the inlet). The cells were cultivated in a minimal salts medium supplied with glucose for 12 h. The mean and standard deviation of the two independent cultures are shown. Note the logarithmic scale in biomass and luminescence y-axes. Total biomass – line with squares; proportion of ABlux cells in total biomass – columns.

For a functional community, the compatibility of the two strains is evidently essential. In order to monitor the performance and growth of ADP1Δ*gntT::Kan^r^/tdk* in the coculture, pVKK81-T-*lux* containing a bacterial luciferase genes *luxCDABE* (from *P. luminescens* [33]) were transformed into the strain by natural transformation. A luminescent colony was selected from a plate containing 10 µg/ml tetracycline. The resulting strain ADP1Δ*gntT*::*Kan^r^/tdk[lux_tet^r^]* was designated as ABlux. In order to monitor the growth dynamics of *E. coli* and ABlux, the strains were cultivated both separately and in cocultures for 12 h in batch cultures. A minimal salts medium MA/9 supplemented with 50 mM glucose was used in the experiment. Biomass and luminescence were determined from the cultivations hourly. Selective plate count was also applied in order to determine the individual cell numbers of *E. coli* and ABlux. The total biomass of the coculture and the proportion of ABlux of the total biomass are presented in Figure 2. The use of luminescent construct enabled specific real-time monitoring of ABlux performance during the cocultivation (Figure 2, the inlet), and allowed the observation of potential fluctuations and problem situations in the cultures, not readily detectable by other means. According to the luminescence signal data, ABlux starts to grow in the coculture after a short lag phase, which was subsequently confirmed by the 5-hour timepoint plate count. After 11 h of cultivation, the strain reached an approximately 6% proportion of the total biomass.

**Figure 3:**
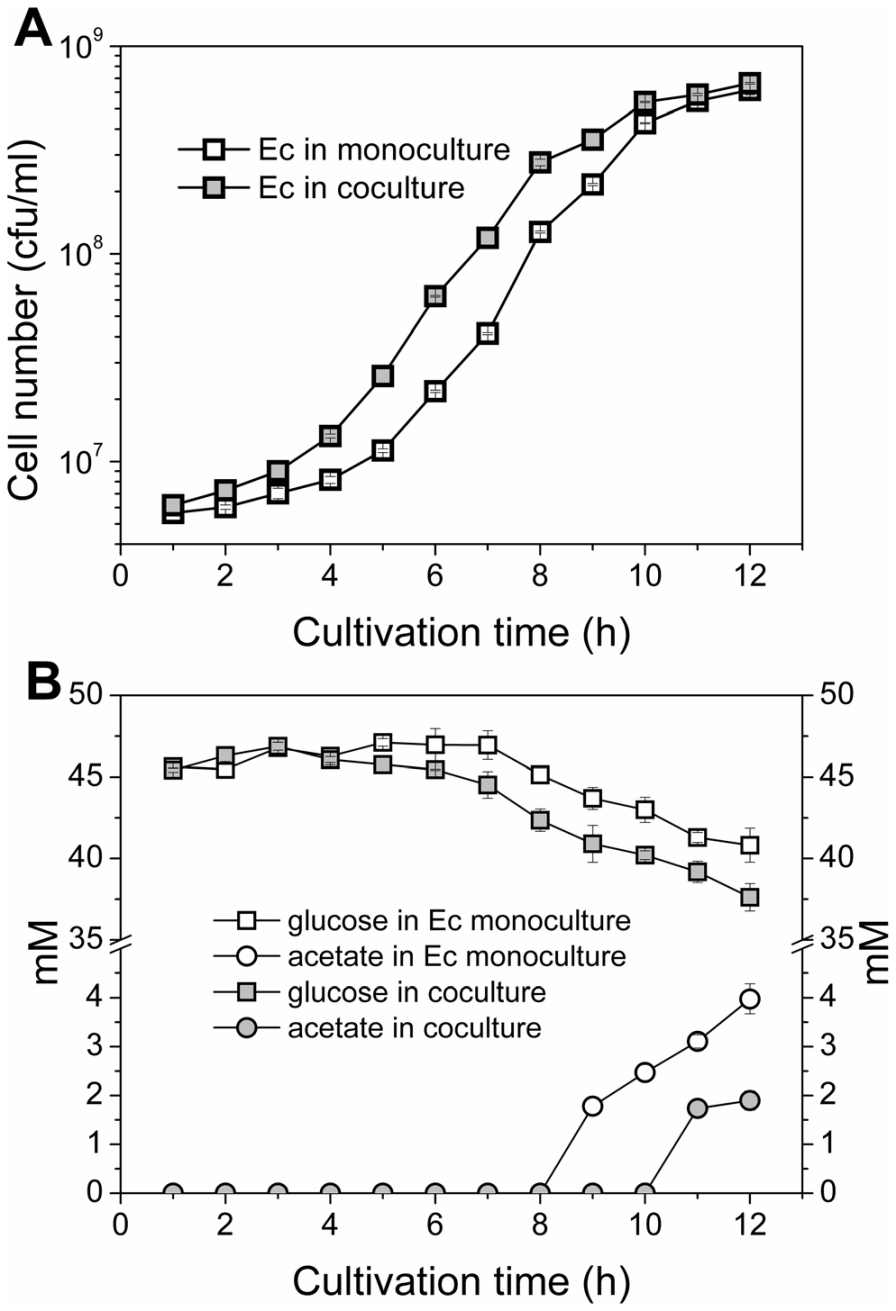
*E. coli* biomasses and substrate/end-metabolite concentrations. The cells were cultivated in a minimal salts medium supplied with glucose for 12 h. A) *E. coli* cell number in monoculture (empty squares) and in coculture (filled squares). B) Glucose (squares) and acetate (circles) concentrations in monoculture (empty symbols) and in coculture (filled symbols). The mean and standard deviation of two independent culture samples are shown. Note the logarithmic scale in biomass y-axis.

The biomass of *E. coli* in the monoculture and in the coculture was calculated according to optical densities and plate counts: the data is presented in Figure 3A. In the *E. coli* monoculture, the growth shifted to an exponential phase after 5–6 hours of lag phase and reached a total cell number of ∼6.15·10^8^ ml^−1^ after 12 h of cultivation. In the coculture, the lag phase for *E. coli* was shorter, 4–5 h, and the culture reached a slightly higher cell number of 6.63·10^8^ ml^−1^ at 12 h timepoint.

The consumption of glucose and accumulation of end-metabolites in *E. coli* monoculture and coculture were analyzed by HPLC (Figure 3B). The consumption of glucose in *E. coli* monoculture was nearly linear after 7 h of cultivation, with a total decrease of 6 mM in the glucose concentration. Acetate could be reliably detected after an 8 h timepoint, the concentration reaching 4 mM at the end of the cultivation. End-metabolites other than acetate were not detected. In the coculture, the glucose was utilized more rapidly and efficiently, with the total decrease in glucose concentration being 9 mM. Detectable amounts of acetate could not be observed in the coculture until the 11 h timepoint, indicating efficient acetate utilization by ABlux. The acetate concentration in the end of the cultivation (2 mM) was lower compared to that of the monoculture of *E. coli*. The strain ABlux did not exhibit growth in the monoculture due to the lack of suitable carbon source, and neither consumption of glucose nor accumulation of end-metabolites was detected (data not shown).

We discovered that the coculture was beneficial for *E. coli*, as the cells grew faster in the cocultivation compared to the monoculture in the studied conditions. As expected, the proportion of ADP1 cells in the coculture increased as a result of *E. coli* acetate production. According to the *E. coli* growth curve, the consumption of acetate by ABlux positively affected the *E. coli* growth, which could be observed as a shorter lag phase and more sufficient growth. Even though the concentration of acetate did not reach the detection limit until the 8–10 h timepoint the cultures already showed differential performance after the 5 h timepoint, indicating that acetate already hinders growth at very low concentrations. Eventually, the coculture resulted in more efficient glucose utilization by *E. coli* and a lower overall acetate concentration in the culture. Thus, it could be concluded that the constructed carbon channeling system diminished the negative effects of acetate and favorably affected the *E. coli* growth, and a relatively low proportion of the supporting strain ABlux in the community was able to benefit the growth.

### Production of recombinant protein by *E. coli* in the synthetic coculture

**Figure 4:**
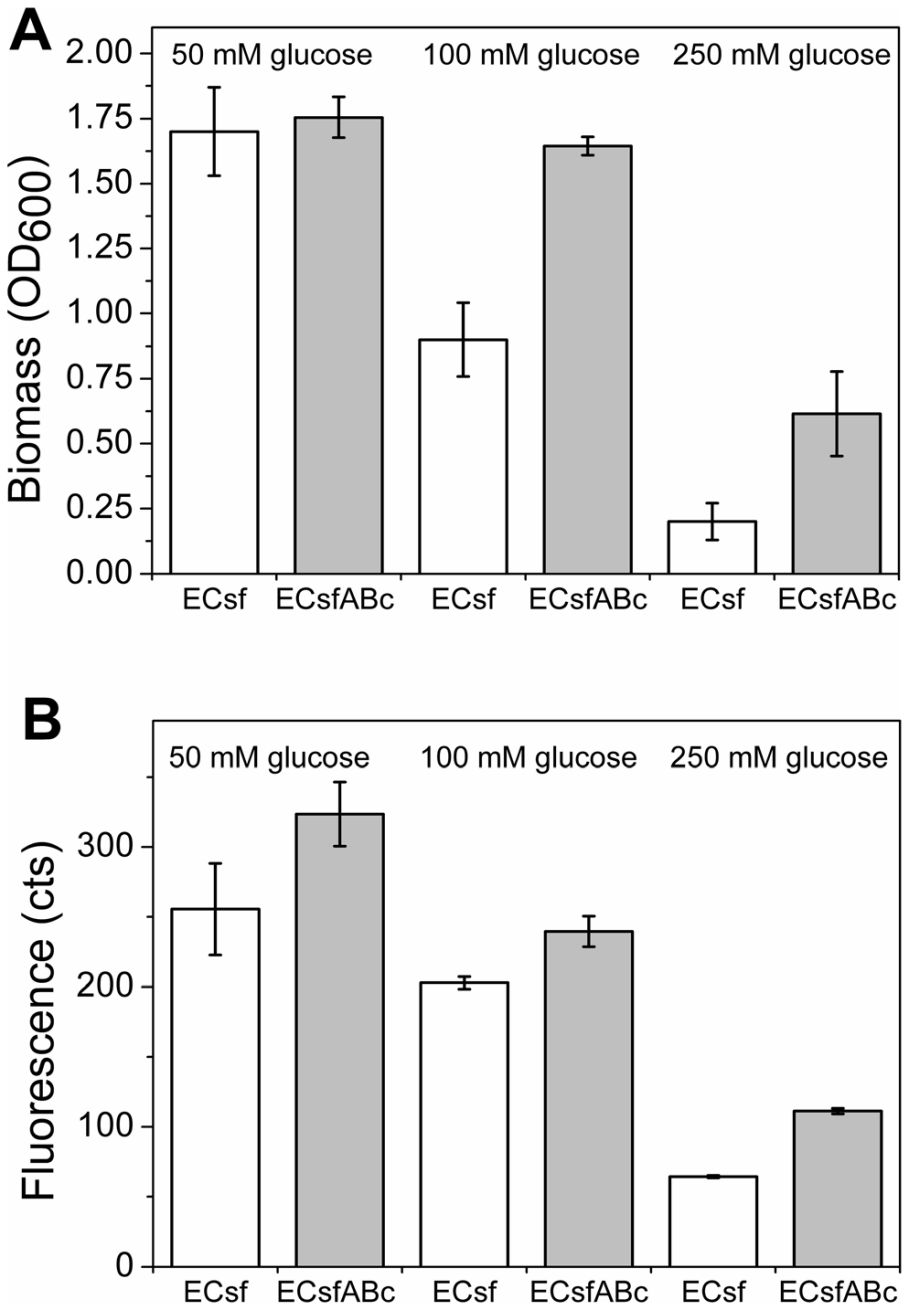
Biomasses and fluorescence of *E. coli* expressing sfGFP/pAK400c (ECsf) and *A. baylyi* ADP1Δ*gntT::Kan^r^/tdk* expressing empty plasmid pBAV1C-*ara* (ABc) in mono and cocultures. Fluorescence is produced solely by *E. coli* expressing GFP. The strains were cultivated in a minimal salts medium supplemented with 50; 100; or 250; mM glucose for 24 h. A) Biomasses (optical density, OD_600_) of ECsf monocultures and cocultures (ECsfABc). B) Fluorescence signals measured for monocultures and cocultures demonstrating the production of recombinant protein in cultures. The mean and standard deviation of two independent cultures are shown.

It was demonstrated that the coculture is beneficial for *E. coli* growth and biomass production. The next step was to study the effects of the coculture on production of the heterologous product that competes with the cell building blocks, demonstrated here by recombinant protein production. Green fluorescent protein (GFP) was chosen for the proof-of-principle production platform in order to enable real-time monitoring of production dynamics and comparability between the mono and cocultures. In addition, GFP has previously been successfully expressed both in *E. coli* and *A. baylyi* ADP1 [21]. A plasmid containing a superfolder variant of GFP (BBa_I746909) under inducible lactose promoter was constructed. The resulting plasmid sfGFP/pAK400c was transformed to *E. coli,* the clones were selected from chloramphenicol plates, and the strain was designated as ECsf. To enable *A. baylyi* ADP1 growth in the coculture, plasmid pBAV1C-*ara* (described elsewhere [22]) without an insert was transformed to the mutant strain ADP1Δ*gntT::Kan^r^/tdk* and selected from the chloramphenicol plates, resulting in a strain designated as ABc. The potential support of ABc for ECsf growth and protein production in a coculture was studied in minimal medium batch cultures supplied with different glucose concentrations: 50 mM, 100 mM, and 250 mM. The amount of GFP produced was estimated by fluorescence determination. The end-point biomasses (OD_600_) and fluorescence signals for monocultures and cocultures are presented in Figure 4. The cocultures grew to higher optical densities and produced more fluorescence with all studied glucose concentrations compared to the respective monocultures, and the highest optical density and fluorescence signal were obtained in the culture containing 50 mM glucose. Interestingly, the more glucose is present in the medium, the more significant is the difference between the mono- and cocultures. This indicates that high substrate concentrations may hinder *E. coli* growth by direct substrate inhibition and by accelerated overflow metabolism. In the cultures containing 250 mM glucose, a fluorescence signal of approximately 2-fold and an optical density of 3-fold higher were obtained for the coculture compared to the monoculture. Thus, the batch experiments further emphasized the coculture relevance for improved culture performance and productivity in variable substrate concentrations.

### Production of recombinant protein by the synthetic coculture in a bioreactor

The previous experiments demonstrated that ABc efficiently supports ECsf growth in cocultures, being especially beneficial in cultures containing high substrate concentrations by neglecting the negative effects of acetate. In an ideal case, the secreted acetate could be directed to the product by the supportive strain. For such an approach to be practical, a single expression construct that is functional in both strains should be used. Indeed, an expression vector exploitable both in *E. coli* and *A. baylyi* ADP1 have been described [21]. Derived from the original vector, a plasmid pBAV1C-T5-GFP was constructed and transformed to *E. coli* and *A. baylyi* ADP1Δ*gntT*::*Kan^r^/*tdk. The vector contains the gene *gfp* under a constitutive strong promoter (T5), and a chloramphenicol resistance gene (*cat*). The resulting strains were designated as ECg and ABg, respectively.

**Figure 5:**
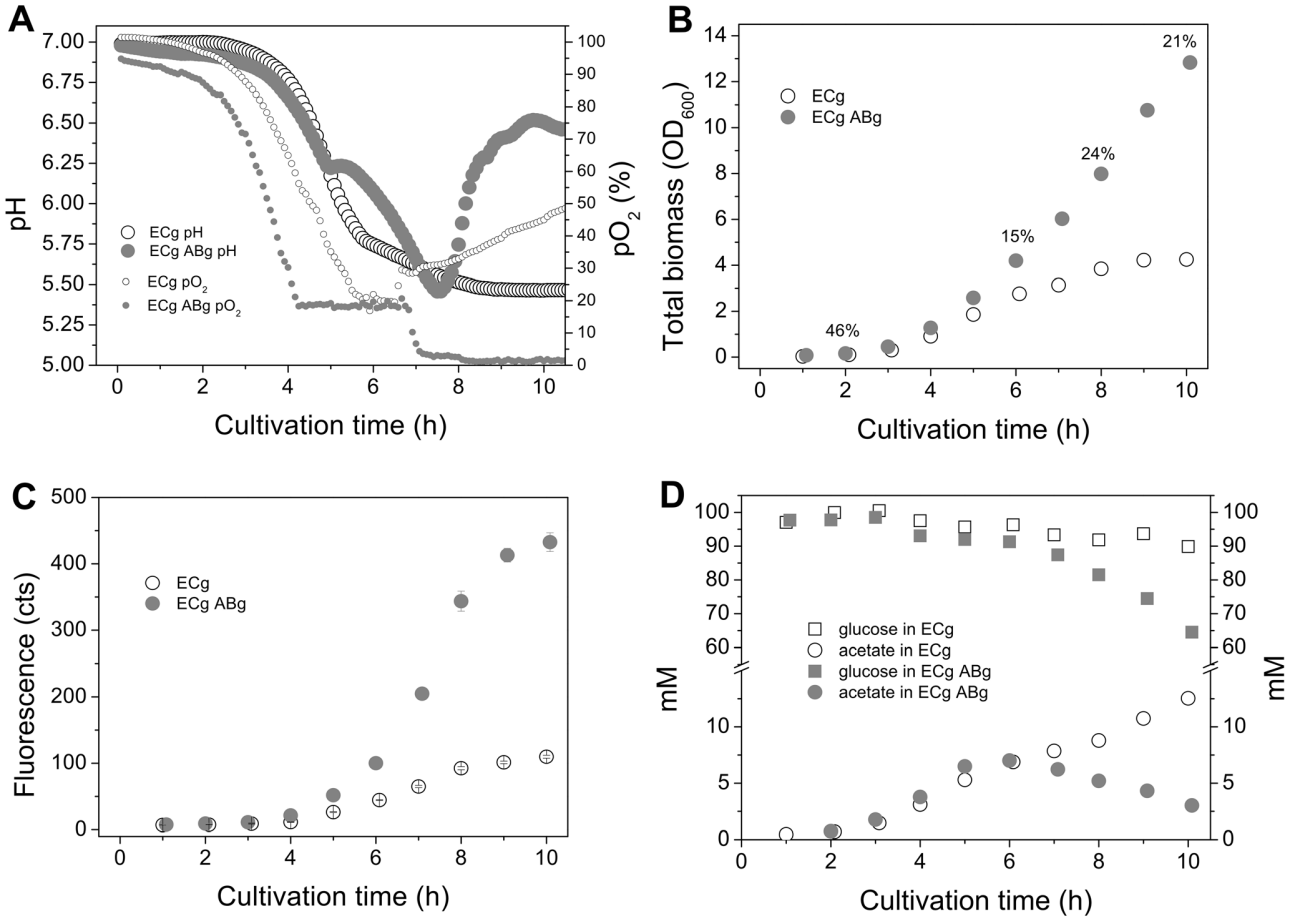
Monitored bioreactor cultivations of the monoculture ECg and the coculture ECgABg. Both strains produce GFP by expressing the same genetic construct pBAV1C-T5-GFP. The cultivations were performed in a bioreactor in a rich medium supplied with 100 mM glucose and aeration. A) Culture parameters; pH (large circles) and oxygen partial pressure (pO2 %, small circles) for ECg monoculture (empty circles) and ECgABg coculture (filled circles) B) Total biomasses (OD_600_) of the cultures and proportion of ABg cells (%, CFU) in the coculture. C) Fluorescence signals (cts) for the monoculture ECg and coculture ECgABg demonstrating the production of recombinant protein in the cultures. The mean and standard deviation of 2–4 independent culture samples are shown. ECg - *E. coli* expressing pBAV1C-T5-GFP, ABg - *A. baylyi ADP1ΔgntT::Kan^r^/tdk* expressing pBAV1C-T5-GFP.

To provide sufficient supply of nutrients and building blocks for the strains under strong over-expression, and to study the coculture functionality in simple unoptimized batch conditions without pH buffering or control, cultivations were performed in a rich medium supplied with glucose. The batch cultivations for the ECg and ABg coculture and ECg monoculture were carried out in a rich medium supplied with 100 mM glucose and aeration. The cultivations were conducted in a bioreactor in order to reveal more information about the culture characteristics and dynamics; oxygen partial pressure, pH, biomass production (OD), proportions of the coculture strains (%, CFU), substrate and end-metabolite concentrations, and fluorescence signals were monitored throughout the 10-hour cultivations (Figure 5). The coculture started to grow clearly faster after four hours of cultivation, reaching a final optical density of 13, which was ∼3-fold higher compared to the biomass of ECg monoculture (OD 4.3). The initial proportions of the two strains were equal but the proportion of ABg seemed to increase fastest between the 6–8 h timepoints, assumingly in parallel with the exponential growth phase of ECg. It was noted that when cultured in a rich medium, the proportion of ABg cells (20–30%) was much higher compared to the minimal medium coculture where the proportion of ABlux was approximately 6% by the end of the cultivation. The relatively high biomass of ABg did not, however, negatively affect ECg growth, which further supports the hypothesis of ADP1 not producing compounds inhibiting *E. coli* growth.

The fluorescence signals differentiated after 4 hours of cultivation, the final fluorescence signals being approximately 4-fold higher in the coculture compared to the ECg monoculture. The higher biomass and consequently the higher fluorescence signal of the coculture can be explained by the more propitious growth conditions; in both cultures, pH started to drop after 3–4 hours of cultivation, but the decline of pH in ECg monoculture was faster compared to the coculture. In the monoculture, the final pH of 5.4 was reached at 8–9 h timepoint. In the coculture, pH decreased more moderately, but increased rapidly after the 8 h timepoint, reaching a final pH of 6.5, which is very close to the initial pH of the culture. This implies an efficient internal buffering system in the coculture, which retains favorable growth conditions.

Acetate concentrations started to differentiate after the 6 h timepoint. At the end of the cultivation, the acetate concentration in the ECg monoculture was 13 mM, which probably affected the growth and cell performance negatively. In the coculture, the acetate concentration was only 3 mM, indicating successful recycle of acetate from the medium to the biomass and the product. The final glucose concentrations were 65 mM in the coculture and 90 mM in the monoculture. For the biomass samples collected at the end of the cultivations, the obtained CDWs were 2.1 g/l and 5.1 g/l for ECg and ECgABg cultures, respectively. Control cultivations without glucose supplementation were carried out in batch bottles. It was found that the coculture does not grow sufficiently in such conditions (data not shown), suggesting that the relevance of the coculture lies for the most part in the interactive glucose-acetate metabolism.

The previous minimal medium cultivations constituted of ECsf and ABc strains demonstrated that both biomass and product titers could be improved, even though ABc was not contributing to the protein production. In a minimal medium, ABc is completely dependent on the carbon provided by ECsf growth, and the proportion of ABc cells is low, whereas rich medium enables more rapid and independent growth of ABc (as for ABg), resulting in significantly higher cell proportions consuming valuable building blocks from protein production. Thus, we were interested to see how the increased number of ABc cells affects the protein production in a rich medium when only ECg contributes to protein production (Figure S1). In short, it was observed that the bioprocess trends, acetate production, glucose consumption, and biomass production were very similar compared to the ECgABg bioreactor coculture. According to the fluorescence signals, the recombinant protein titer was 50% higher in the ECgABc coculture compared to the ECg monoculture, which is expectedly less than improvements gained in ECgABg coculture. It can be concluded that as protein overexpression is highly dependent on the available biocomponents consumed by both the strains, it is not energetically affordable to maintain the system in a rich medium with high proportions of the supportive strain, without it participating in the production. Thus, the availability of genetic tools and the possibility to rationally engineer and commit both the strains to the production are crucial to take full advantage of the carbon redirection, as demonstrated here. Furthermore, the product example (recombinant protein) is constituted of amino acids instead of carbon based molecules, and thus the benefits of carbon rerouting could be potentially better exploited in production of hydrocarbons, such as fatty acid derived compounds for bioenergy [22], [23]. Our findings suggest it is possible to tune the coculture balance, nature, and dynamics by altering the medium, substrate concentration, and genetic constructs.

The accumulation of acetate is a widely recognized problem in bioprocessing of *E. coli*. This study demonstrates that by exploiting a coculture with optimally chosen strains, the growth, carbon utilization, and product formation can be improved through symbiotic interactions, and the negative effects of acetate can be diminished. Employing the coculture enabled the use of a high substrate concentration in a simple batch culture, and the over-flow metabolism of *E. coli* was successfully exploited in producing the protein of interest. Fluctuations in sugar concentrations in industrial feedstocks can impose restrictions for single strain cultures (6), whereas high substrate concentration is not an issue for the coculture system presented here. Thus, with regards to sustainable and economical carbon sources, exploiting a well-designed coculture may broaden the possibilities to exploit challenging liquors with high concentrations of carbohydrates.

Nevertheless, cocultures are complex systems involving numerous interactions. Despite the possibility to reduce these interactions, it is very difficult to identify all the mechanisms that are potentially beneficial for the culture. *E. coli* based bioprocesses have demanded decades of optimization work to build up a cost-effective bioproduction platform [34]. Thus, improvement of the utility, profitability, and viability of cocultures requires significant research efforts. It is impossible to exceed the production rates and yields of highly tuned and optimized systems at a single cell level, but the power of cocultures most probably lie in processes that cannot be readily optimized (e.g. processes exploiting diverse industrial streams) or in processes involving variable conditions.

## Conclusions

Synthetic biology broadens the possibilities to tune sophisticated production platforms, and coculturing is seen as a promising new frontier for taking the bioproduction to the next level [35], [36]. This study demonstrates that rationally engineered synthetic cocultures can improve biomass production, culture viability, and product formation in simple unoptimized batch conditions. The study extends applications of novel type of bioprocess optimization, and provides clues for the development of functional, readily engineered, and dynamic cocultures for synthetic biology applications.

## Supporting Information

Figure S1 Bioreactor data of ECg and ABc coculture.
(PDF)
