# Supplementary material for: Rationally Engineered Synthetic Coculture for Improved Biomass and Product Formation
Source: PLoS One. 2014 Dec 3;9(12):e113786. doi: 10.1371/journal.pone.0113786 (PMC4254613; doi:10.1371/journal.pone.0113786)
Supplement: Figure S1 — Bioreactor data of ECg and ABc coculture. (PDF) [file pone.0113786.s001.pdf]

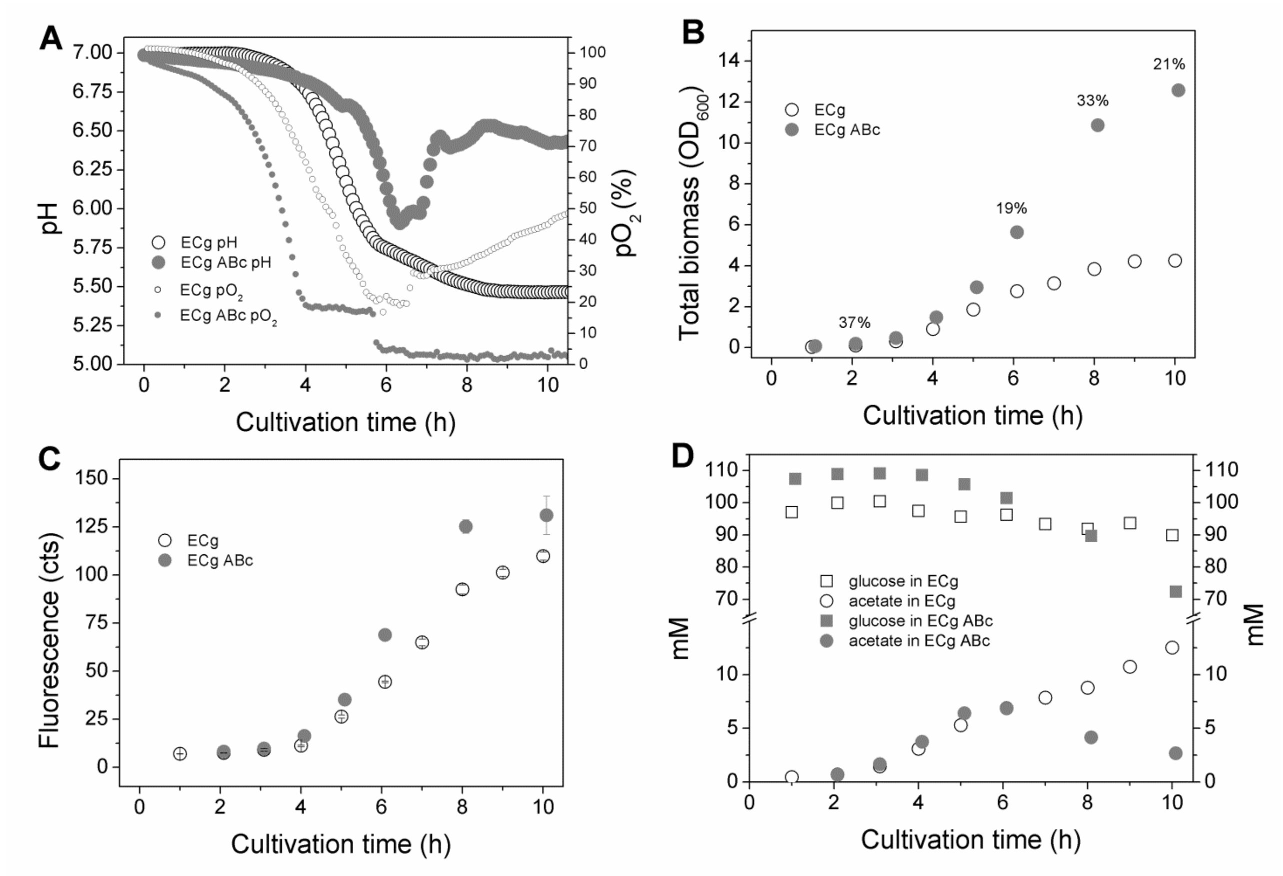

Figure S1. Bioreactor data of ECg and ABc coculture. A coculture of *E. coli* expressing GFP (strain ECg) and the non-fluorescent control strain *Acinetobacter baylyi*ADP1Δ*gntT*::*Kan*^{r}*/tdk.* (strain ABc) was carried out in a bioreactor in a rich medium supplied with 100 mM glucose and aeration. ECg is the only strain contributing to the fluorescence production. The monoculture of ECg is presented for a reference. A) Culture parameters; pH (large circles) and oxygen partial pressure (pO2 %, small circles) for ECg monoculture (empty circles) and ECgABc coculture (filled circles) B) Total biomasses (OD_{600}) of the cultures and the proportion of ABc cells (%, CFU) in the coculture. C) Fluorescence signals (cts) for the monoculture ECg and the coculture ECgABc demonstrate the production of recombinant protein in the cultures. The mean and standard deviation of 2-4 independent culture samples are shown. ECg - *E. coli* expressing pBAV1C-T5-GFP, ABc - *A. baylyi* ADP1Δ*gntT::Kan*^{r}*/tdk* expressing pBAV1C-*ara* without insert.
